# Differences in men and women suffering from CRSwNP and AERD in quality of life

**DOI:** 10.1007/s00405-020-06418-5

**Published:** 2020-10-15

**Authors:** Tina J. Bartosik, David T. Liu, Nicholas J. Campion, Sergio Villazala-Merino, Stefan Janik, Valerie Dahm, Christian A. Mueller, Erich Vyskocil, Victoria Stanek, Tamara Quint, Christine Bangert, Julia Eckl-Dorna, Sven Schneider

**Affiliations:** 1 Department of Otorhinolaryngology, Medical University of Vienna, Währinger Gürtel 18-20, 1090 Vienna, Austria; 2 Department of Dermatology, Medical University of Vienna, Währinger Gürtel 18-20, 1090 Vienna, Austria

**Keywords:** Chronic rhinosinusitis, Sex, Polyps, Aspirin-exacerbated respiratory disease, AERD, Samter’s triad, SNOT-20 GAV

## Abstract

**Purpose:**

While the overall impact of chronic rhinosinusitis (CRS) on patients’ health is diverse, many affected individuals have a substantially impaired quality of life (QoL). The aim of this study was to evaluate the impact of sex-associated differences specifically in the subgroups of CRS with nasal polyps (CRSwNP) and aspirin-exacerbated respiratory disease (AERD) by assessing QoL parameters in women and men separately.

**Methods:**

In a retrospective single-center study, 59 patients with CRSwNP (39 males and 20 females) and 46 patients with AERD (18 males and 28 females) were included. Patient-reported outcome measures (PROM) evaluating QoL via the Sino-Nasal Outcome Test-20 German Adapted Version (SNOT-20 GAV) as well as the total polyp score (TPS) were analysed.

**Results:**

There was no significant difference in TPS (*p* = 0.5550) and total SNOT-20 GAV scores (*p* = 0.0726) between male or female patients with CRSwNP or AERD. Furthermore, no significant sex differences were found within disease groups regarding the subcategories of the SNOT-20 GAV items.

**Conclusion:**

Thus, quality of life is severely impaired in patients suffering from various forms of CRS regardless of their sex.

## Introduction

Sex- and gender-related factors are present in a variety of diseases affecting the clinical presentation and treatment of these medical conditions, as well as the impact on patients’ quality of life (QoL) [1]. Among others, sex-related aspects have not only been identified regarding genetics and hormones, but interestingly also when analysing epigenetics, immune function, aging including neurocognitive decline, vascular status as well as response to therapeutics and interaction with the health care system [2].

Chronic rhinosinusitis (CRS) is a common, yet diverse disease affecting 4–16% of the general population [3–5]. According to the recently published current version of the European Position Paper on Rhinosinusitis and Nasal Polyps (EPOS 2020), the condition should be classified into primary and secondary CRS. While the former is associated with generalized inflammation, the latter is caused by other underlying triggers including tumours, odontogenic reasons, or fungal growth. Primary CRS can be further sub-grouped as localized (unilateral) or diffuse (bilateral) disease and phenotypically grouped according to the absence or presence of nasal polyps. Phenotypes are determined by underlying type 2 (CRS with nasal polyps, CRSwNP) or non-type 2 inflammation (CRS without nasal polyps, CRSsNP) [6]. CRSwNP affects between 2.7 and 4.4% of the population and approximately 10% of these patients with nasal polyps suffer from aspirin-exacerbated respiratory disease (AERD) syndrome, which is characterized by the clinical triad of nasal polyps, bronchial asthma, and hypersensitivity to non-steroidal anti-inflammatory drugs (NSAIDs) [6–8]. CRSwNP and AERD generate not only nasal symptoms but also impair quality of sleep, mood, cognition, and productivity [9].

Patient-reported outcome measures (PROMs) are important instruments to evaluate the subjective burden of disease. Sino-nasal outcome test-22 (SNOT-22) and the sino-nasal outcome test-20 German adapted version (SNOT-20 GAV) are valid, reliable, and widely used tools to specifically address CRS symptoms [10, 11]. In this study, we focused on the comparison of patients suffering from CRS presenting with nasal polyps (CRSwNP) alone and those diagnosed with AERD.

Studies evaluating sex and gender specific impact on QoL of patients with CRSwNP and AERD are discordant. Furthermore, differences in men and women could also exist in specific phenotypes of CRS. Recently, a cluster analysis of SNOT-22 and VAS values regarding CRSwNP and CRSsNP patients according to gender was performed. Three clusters were identified: the first cluster comprised 37 female patients with CRS without nasal polyps (CRSsNP), the second cluster comprised 30 patients with CRS and NP (CRSwNP; 15 males and 15 females); and the third cluster had 30 male patients with CRS without NP (CRSsNP), indicating similarities in men and women diagnosed with CRSwNP but not CRSsNP patients [12]. With regards to sex as a factor influencing PROMs, higher SNOT-22 scores in patients suffering from CRS (with and without polyps) presenting for sinus surgery have been reported in women [13]. Elaborating on sex-specific differences in CRS, a higher prevalence of facial pain and headache was observed in female patients, whereas nasal obstruction was described more frequently in men [14]. In another study examining QoL of patients with CRS, worse QoL results were found among women in spite of similarities in objective disease measures, which were attributed to the increased prevalence of depression and AERD. When patients with depression or aspirin sensitivity were removed from the analysis, no statistically significant gender differences could be found [15].

Modern therapeutic strategies aim at personalized management of the individual patient. Therefore, knowledge of sex and sex-specific influences might be relevant to improve care for patients suffering from CRSwNP and AERD. Thus, the aim of this study was to clarify the impact of CRSwNP and AERD on patients’ QoL in women and men in order to possibly adapt future therapies accordingly.

## Materials and methods

### Study population

The protocol was approved by the ethics committee of the Medical University of Vienna (EK 1630/2019) and the study was conducted according to the guidelines of the Declaration of Helsinki on Biomedical Research Involving Human Subjects. Patients with nasal polyps (aged 18 and older) presenting at the Department of Otorhinolaryngology and the Department of Dermatology at the Medical University of Vienna between 2017 and 2019 for further therapy were included and had to provide written informed consent. Subjects suffering from vasculitis, cystic fibrosis, odontogenic sinusitis, fungal sinusitis, sarcoidosis, and autoimmune disease (secondary CRS) were excluded.

During the visit, patients’ sex and age were recorded. Patients were asked to complete a questionnaire including the SNOT-20 GAV and nasal polyposis was assessed via endoscopy by a trained otorhinolaryngologist. CRS was diagnosed based upon the criteria according to the EPOS 2020 [6]. Furthermore, the diagnosis of AERD was defined according to the European Academy of Allergy and Clinical Immunology (EAACI) position paper as the documented presence of nasal polyps and asthma, in addition to developing respiratory symptoms upon the ingestion of aspirin or other non-steroidal anti-inflammatory drugs (NSAID) [16]. If nasal polyps were present at any time in a patient’s medical history, he or she was classified as CRSwNP. Consequently, patients’ diagnoses remained CRSwNP or AERD even if nasal polyps were not present at the time of the study visit due to recent surgical removal.

### Outcome measures

To grade the nasal polyp size by nasal endoscopy, the total polyp score (TPS) system was used [17]. Both sides of the nasal cavity were separately assessed and scored based on polyp size, resulting in scores of 0–4 (0 = no polyps, 1 = small polyps in the middle meatus not reaching below the inferior border of the middle turbinate, 2 = polyps reaching below the lower border of the middle turbinate, 3 = large polyps reaching the lower border of the inferior turbinate or polyps medial to the middle turbinate, 4 = large polyps causing complete obstruction of the inferior nasal cavity) [18]. The sum of both nostril scores was considered as the TPS.

The SNOT-20 GAV, was used to record QoL [10, 19]. SNOT-20 GAV is a reliable, validated and sensitive German instrument for measuring health-related QOL in patients with CRS [20]. SNOT- 20 GAV differs from the widely used version in English SNOT-22 as the items “need to blow nose”, “lack of good night’s sleep” and “fatigue” are not represented in SNOT-20 GAV, but additional item “need to clear throat/dry throat” is included. The parameters of the test are graded from 0 to 5 (0 = no problem, 1 = very mild problem, 2 = mild or slight problem, 3 = moderate problem, 4 = severe problem, 5 = problem as bad as can be). Moreover, the 20 questions were summarized in four subcategories: nasal symptoms (“nasal blockage”, “sneezing”, “runny nose”, “post-nasal discharge”, “thick nasal discharge”, “need to clear throat/dry throat”, “cough”, “sense of smell”), otologic symptoms (“ear congestion”, “ear pain”, “dizziness”, “facial pain/pressure”), sleep symptoms (“difficulty falling asleep”, “waking up at night”, “fatigued or tired during the day”, “reduced productivity”, “reduced concentration”, “frustration, restlessness, irritability”) and emotional symptoms (“sad”, “embarrassed”). Each category, as well as each symptom, was analysed separately.

### Statistical analysis

Study data were collected and managed using Microsoft Excel for iOS 16.25 (Microsoft Corporation, Washington, USA). GraphPad Prism 8.4.2 (GraphPad Software, Inc., La Jolla, San Diego, CA, USA) was used for statistical analysis and visualization of the results. Normality of data was tested using Kolmogorov–Smirnov test with Dallal Wilkinson-Lille being used for calculation of the *p* value.

The patient characteristics like age, TPS, number of previous surgeries, and the SNOT score were described as mean (± standard deviation). Welch’s ANOVA was used to compare group differences and followed by Dunnett’s T3 multiple comparisons test.

A *p* value of < 0.05 was required for statistical significance.

## Results

### Patient characteristics

**Fig. 1:**
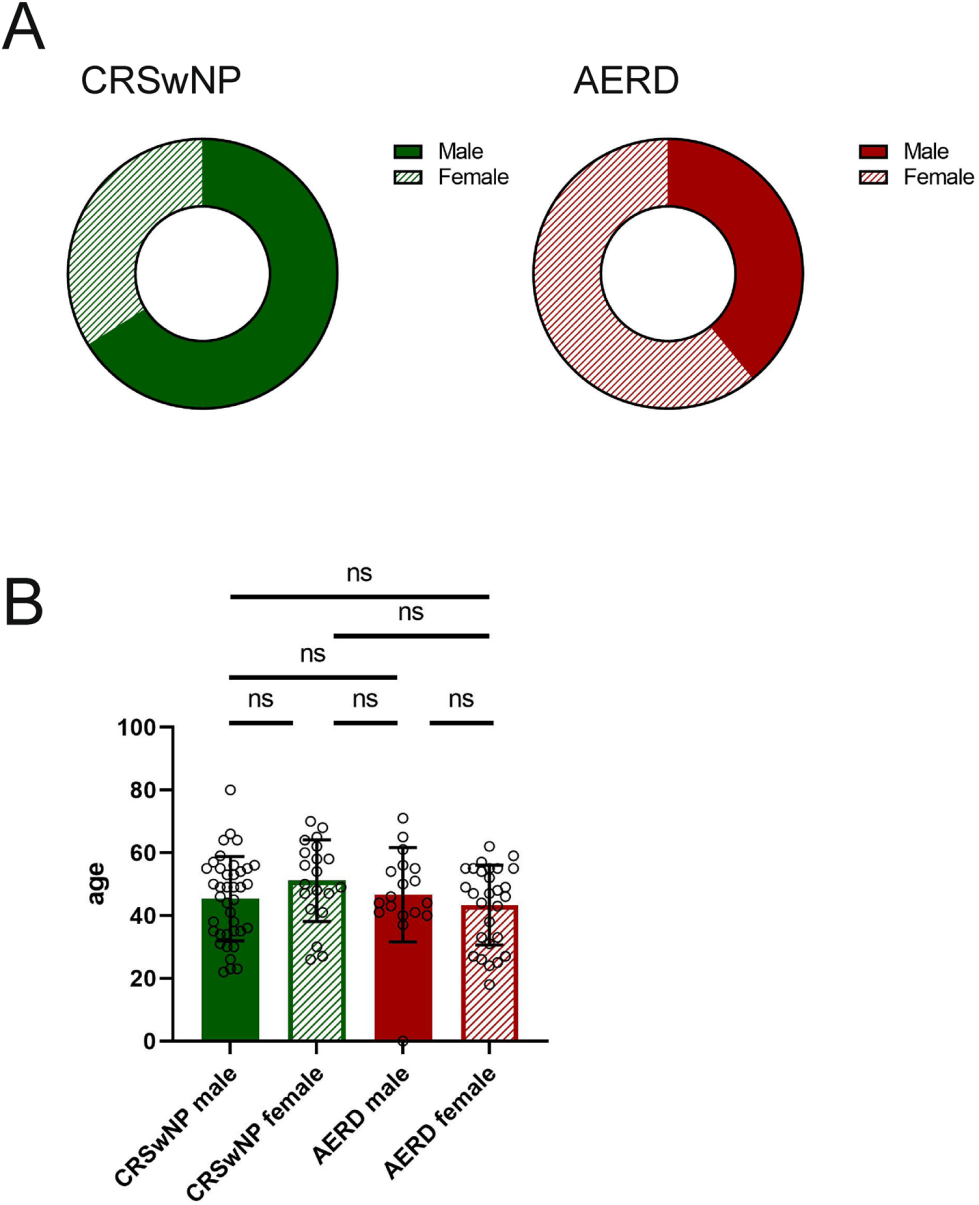
Sex and age distribution in patients suffering from chronic rhinosinusitis with nasal polyps (CRSwNP, green) or aspirin-exacerbated respiratory disease (AERD, red). **a** Sex distribution in patients with CRSwNP or AERD. **b** Average age (*y*-axis, in years) in the two patient groups divided by sex (*x*-axis). Bars represent mean values with standard deviation (SD). Filled bars represent male patients, striped bars represent female patients. No significant differences were observed between the groups (Welch’s ANOVA *p* = 0.2591). *ns* not significant

A total of 105 patients with nasal polyps presenting to the outpatient clinic of the Department of Otorhinolaryngology or the Department of Dermatology at the Medical University of Vienna were included in this study. Among patients suffering from isolated CRSwNP, 66% (*n* = 39) were male and 34% (*n* = 20) were female. 39% (*n* = 18) of the AERD patients were male and 61% (*n* = 28) female. Patient details are depicted in Table 1 and Fig. 1a.

**Table 1:** Patients characteristics

	CRSwNP male (*n* = 39)		CRSwNP female (*n* = 20)		AERD male (*n* = 18)		AERD female (*n* = 28)	
	Mean	SD	Mean	SD	Mean	SD	Mean	SD
Age	45.36	13.41	51.10	13.01	46.61	15.00	43.36	12.69
TPS	3.87	2.27	3.40	1.57	4.44	2.99	3.54	2.67
SNOT score	33.91	15.74	40.40	20.01	47.56	21.14	41.79	18.48
No. of surgeries	0.82	0.95	0.75	0.91	3.72	3.32	1.96	1.37

The mean age of patients was 46.6 years and no statistically significant differences in age were observed between the stratified male and female groups (Fig. 1b).

### TPS and history of surgeries in patients suffering from CRSwNP or AERD according to sex

**Fig. 2:**
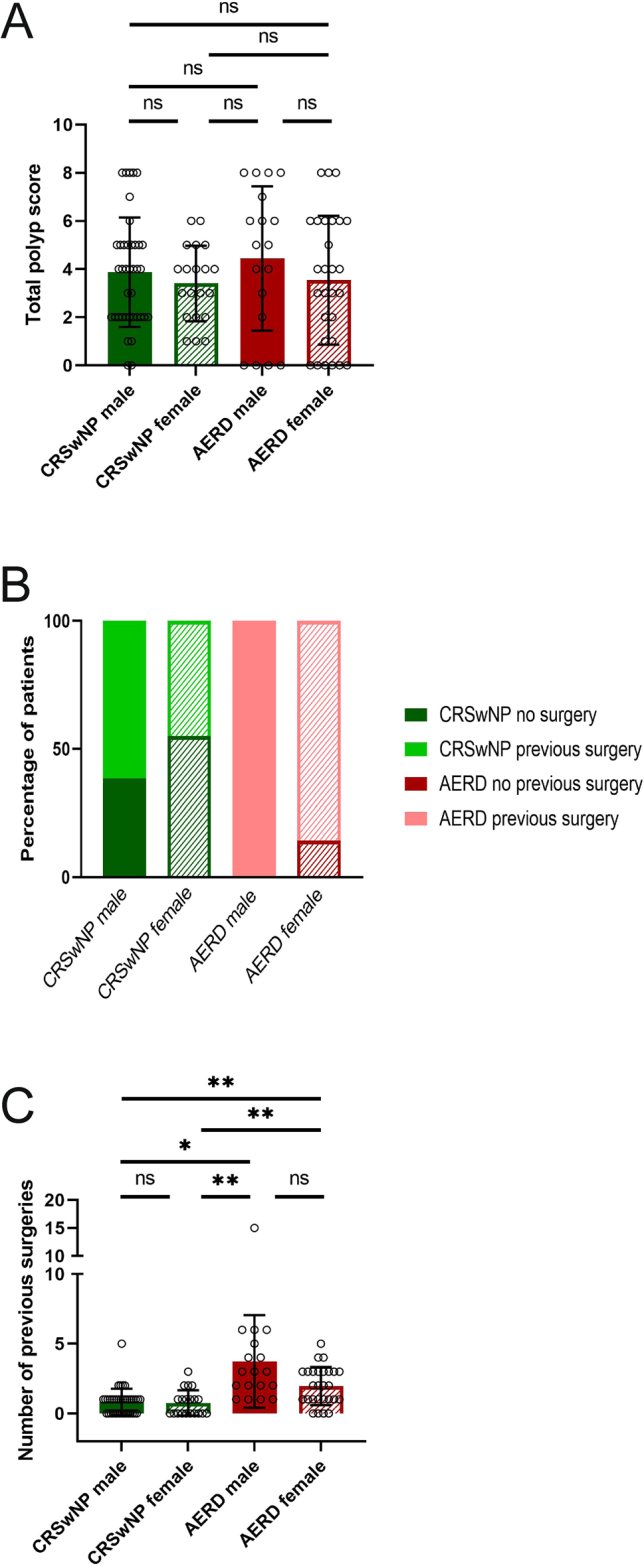
Total polyp score (TPS) and previous surgeries in male (filled bars) and female (striped bars) patients suffering from chronic rhinosinusitis with nasal polyps alone (CRSwNP, green) or aspirin-exacerbated respiratory disease (AERD, red). **a** TPS (*y*-axis) in the two patient groups according to sex (*x*-axis). Bars represent mean values with standard deviation (SD). No significant differences were observed between the groups (Welch’s ANOVA *p* = 0.5550). **b** Percentage of patients who had previous surgery (*y*-axis) in the two patient groups according to sex (*x*-axis). Dark colors represent no prior surgeries whereas light colors signal previous surgeries. **c** Average number of previous surgeries per patient group. Significant differences between patient groups were observed (Welch’s ANOVA: *p* < 0.0001), significant differences in pairwise comparisons using Dunnett’s T3 multiple comparison test were shown between CRSwNP male and AERD male (*p* = 0.0108), CRSwNP male and AERD female (*p* = 0.0031), CRSwNP female and AERD male (*p* = 0.0094), and CRSwNP female and AERD female (*p* = 0.0042).  * *p* ≤ 0.05, **p ≤ 0.01, ***p ≤ 0.001, ****p ≤ 0.0001, *ns* not significant

In the group of patients suffering from CRSwNP alone, the mean TPS was 3.87 (SD = 2.27) in male and 3.40 (SD = 1.57) in female patients (Table 1; Fig. 2a). Male AERD patients scored a mean TPS of 4.44 (SD = 2.99) and females a mean of 3.54 (SD = 2.67). No significant differences in the four subgroups were observed (Welch’s ANOVA *p* = 0.5550).

No significant differences in female and male patients according the number of previous surgeries were observed within the disease groups (Dunnett’s T3 multiple comparison test CRSwNP male vs. female *p* > 0.9999 and AERD male vs. female *p* = 0.2286). Of the CRSwNP patients, 44.93% (*n* = 33) had prior surgery compared to 91.30% (*n* = 42) in the AERD group. The mean number of previous surgeries was 0.82 (SD = 0.95) in male CRSwNP patients, 0.75 (SD = 0.91) in female with CRSwNP, 3.72 (SD = 3.32) in male AERD patients, and 1.96 (SD = 1.37) in female AERD patients (Table 1). On the other hand, significant differences in pairwise comparisons using Dunnett’s T3 multiple comparison test were shown between CRSwNP male and AERD male (*p* = 0.0108), CRSwNP male and AERD female (*p* = 0.0031), CRSwNP female and AERD male (*p* = 0.0094), and CRSwNP female and AERD female (*p* = 0.0042).

### SNOT-20 GAV score in male and female patients suffering from CRSwNP or AERD

In the group of patients suffering from CRSwNP, the mean SNOT-20 GAV score was 33.91 (SD = 15.74) in male and 40.40 (SD = 20.01) in female patients. Male AERD patients scored a mean SNOT-20 GAV score of 47.56 (SD = 21.14) and females a mean of 41.79 (SD = 18.48).

**Fig. 3:**
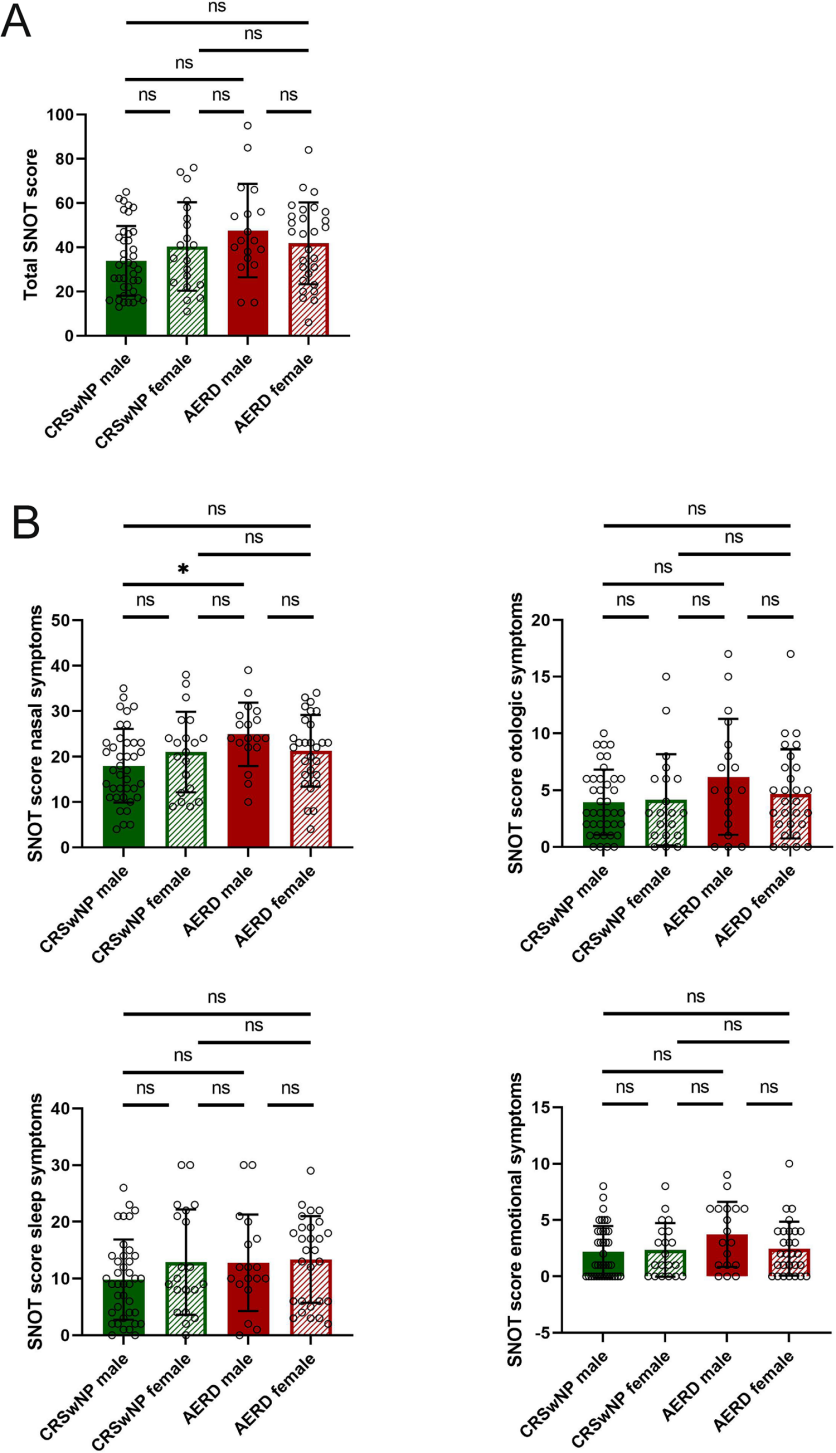
Sino-Nasal Outcome Test-20 German Adapted Version (SNOT-20 GAV) score in patients suffering from chronic rhinosinusitis with nasal polyps (CRSwNP, green) or aspirin-exacerbated respiratory disease (AERD, red). **a** SNOT-20 GAV score (*y*-axis) in the two patient groups displayed by sex (*x*-axis). No significant differences were observed between the groups (Welch’s ANOVA *p* = 0.0726). **b** SNOT-20 GAV grouped into the four categories “nasal symptoms”, “otologic symptoms”, “sleep symptoms”, and “emotional symptoms”. No significant differences were observed except for between the male CRSwNP and AERD patients in the category of “nasal symptoms” (Welch’s ANOVA *p* = 0.0200; Dunnett’s T3 multiple comparison test *p* = 0.0121). Filled bars represent male patients, striped bars female patients. Bars represent mean values with standard deviation (SD). Significance is displayed in figures *p ≤ 0.05, **p ≤ 0.01, ***p ≤ 0.001, ****p ≤ 0.0001, *ns* not significant

No significant differences between male and female patients were observed (Table 1; Fig. 3a). However, when analyzing the four categories of the SNOT—nasal, otologic, sleep and emotional symptoms—male patients with AERD suffered significantly more from “nasal symptoms” as compared to male patients with CRSwNP (Dunnett’s T3 multiple comparison test *p* = 0.0121).

### Single item scores with significant differences between male and female patients suffering from CRSwNP or AERD

**Fig. 4:**
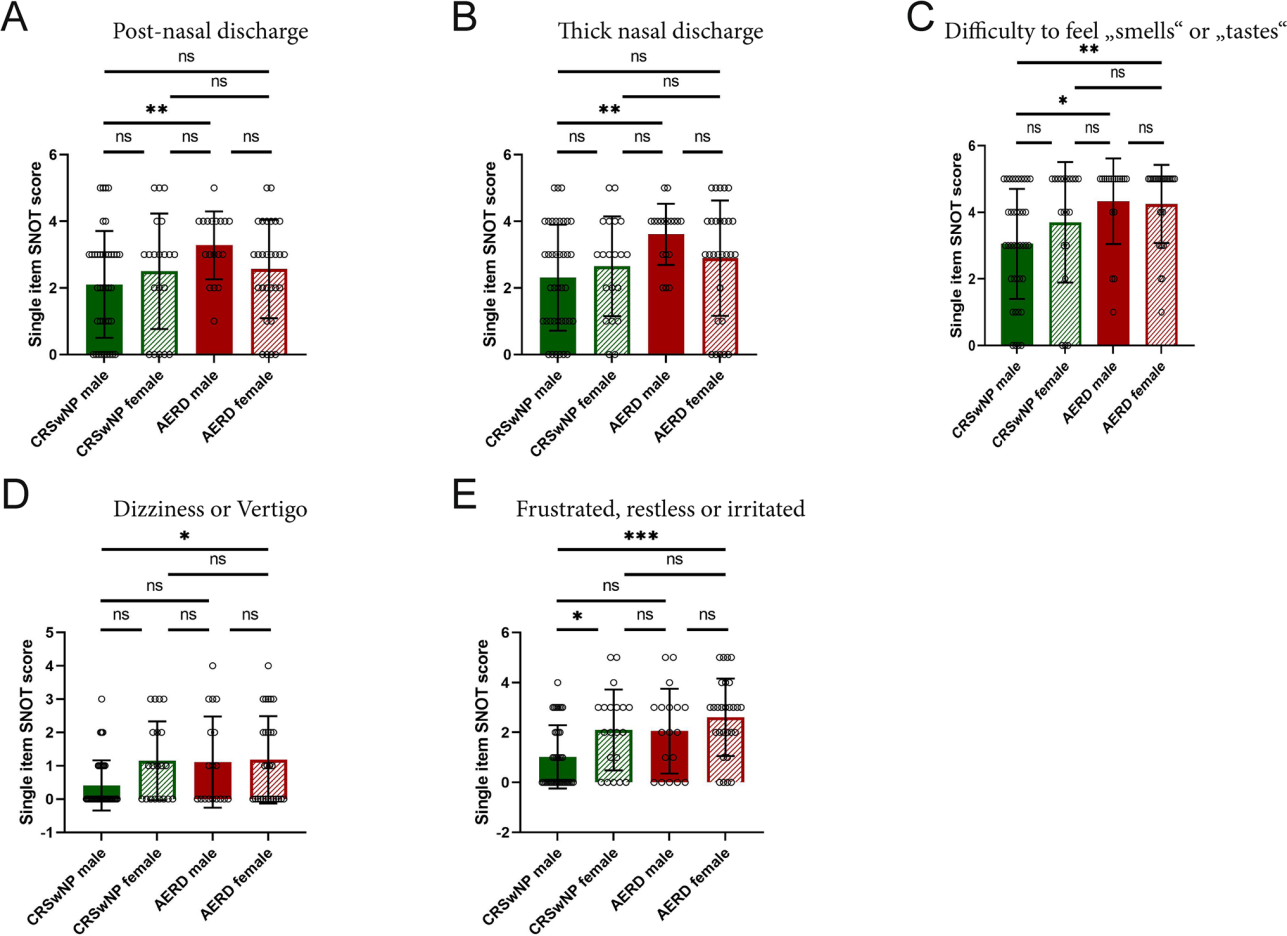
Single item score of SNOT-20 GAV comparison in patients suffering from chronic rhinosinusitis with nasal polyps (CRSwNP, green) and aspirin-exacerbated respiratory disease (AERD, red) (A-E) SNOT-20 GAV score (*y*-axis) in the two patient groups divided by sex (*x*-axis, male = filled bars, female = striped bars) for the following items: **a** Post-nasal discharge (Welch’s ANOVA *p* = 0.0162). **b** Thick nasal discharge (Welch’s ANOVA *p* = 0.0027). **c** Difficulty to feel “smells” or “tastes” (Welch’s ANOVA *p* = 0.0049). **d** Dizziness or Vertigo (Welch’s ANOVA *p* = 0.0076). **e** frustration, restlessness or irritation” (Welch’s ANOVA *p* = 0.0004). Bars represent mean values with standard deviation (SD). Significance is displayed in figures *p ≤ 0.05, **p ≤ 0.01, ***p ≤ 0.001, ****p ≤ 0.0001, *ns *not significant

Next, we analysed the single items of the SNOT-GAV 20 stratified by disease and sex and found no signifcant sex differences within disease groups. However, in the category nasal symptoms, male patients with AERD suffered more from the items post-nasal (Welch’s ANOVA *p* = 0.0162; Dunnett’s T3 multiple comparison test *p* = 0.0094) and thick nasal discharge (Welch’s ANOVA *p* = 0.0027; Dunnett’s T3 multiple comparison test *p* = 0.0017) as compared to their CRSwNP counterparts (Fig. 4a, b). Both male and female patients with AERD scored higher in the item “Difficulty to feel ‘smells’ or “tastes” than male patients with CRSwNP (Fig. 4c; Welch’s ANOVA *p* = 0.0049; Dunnett’s T3 multiple comparison test CRSwNP male vs. AERD male *p* = 0.0159, CRSwNP male vs. AERD female *p* = 0.0056). Females with AERD showed significant differences as compared to CRSwNP male patients with regard to the items “Dizziness or Vertigo” (category otologic symptoms; Welch’s ANOVA *p* = 0.0076; Dunnett’s T3 multiple comparison test CRSwNP male vs. AERD female *p* = 0.0456)(Fig. 4d) and “Frustrated, restless or irritated” (category emotional symptoms; Welch’s ANOVA *p* = 0.0004; Dunnett’s T3 multiple comparison test CRSwNP male vs. AERD female *p* = 0.0003) (Fig. 4e). In all the other items, no significant differences between the four groups were observed (data not shown).

## Discussion

In this study, we examined the association of sex and QoL in patients suffering from CRSwNP and AERD. So far, aspects of gender and sex differences in these entities have been poorly investigated. We conducted this study with a large cohort of AERD (*n* = 46) and CRSwNP patients (*n* = 59). In summary, we found no significant difference in TPS and total SNOT-20 GAV scores between male or female patients with CRSwNP or AERD.

With the aim of achieving precision medicine, aspects of biological sex and the psychosocial aspects of a patient’s gender identity are receiving growing attention in various disease states [21–23]. A wide range of factors including genetics, epigenetics, hormones, immune function, and behaviour might be influenced by patients’ sex and gender identity [2]. CRS is a highly prevalent disease and especially phenotypes with nasal polyps and AERD can significantly impair patients’ QoL [9]. Although the impact of sex in CRSwNP and AERD is not yet fully elucidated, studies evaluating e.g. the nasal microbiome in patients with CRS suggest differences in microbial colonization in women and men [24]. Furthermore, sex and gender specific differences might differ in CRS phenotypes like CRsNP, CRSwNP patients and patients suffering from AERD. A recently published cluster analysis evaluating QoL of CRS patients displayed three groups with similar symptoms. One cluster comprised women with CRSsNP, another cluster grouped men with CRSwNP and the third cluster represented women and men diagnosed with CRSwNP [12].

The QoL and impairment of olfactory function in AERD patients has been confirmed by previous research [25, 26], but interestingly, AERD and especially severe cases are more prevalent amongst women [27]. It has been reported that women and men can perceive and report symptoms and burden of disease differently [28]. It was considered that women are more likely to report their symptoms and give a worse assessment of their health. However, the extent of the sex differences in health also vary according to the particular symptom or condition and to the phase of life [29]. It could, therefore, be hypothesized that studies in CRS, which assess the subjective complaints by the participants, would show a lower QoL among female patients.

Interestingly, we did not observe any significant differences in QoL between male and female patients diagnosed with CRSwNP and AERD. Similarly, Mendolia-Loffredo et al. also found no difference in disease-specific health-related QoL between sexes of patients with CRS [15, 30]. In this study analysis of the whole cohort revealed that females seemed to score worse than males on QoL scores. However, when further analysing the data, the authors noted that the poorer outcome was caused by the higher prevalence of acetylsalicylic acid intolerance and depression among women. Consequently, differences in QoL according to sex were eliminated after exclusion of patients with these comorbidities [15]. These results are in line with our observation where we could not find any major differences in QoL scores between male and females regardless whether they suffered from CRSwNP or AERD. It is, therefore, conceivable that the influence of sex is restricted primarily to the general QoL and that symptoms such as migraine headache, which are more common among women, and factors such as difference in anatomic size and hormone increases the prevalence of CRS in women compared to men as suggested by Ference et al. [30].

In the analysis of SNOT-20 GAV single items, we found the highest scores and significant differences in the symptom parameters “thick nasal discharge”, “post-nasal discharge”, and “difficulty to smell or taste” between the groups with CRSwNP and AERD in male patients only. In line with these results, Schneider et al. also observed higher SNOT-20 GAV scores in AERD patients as compared to CRSwNP especially in the category nasal symptoms [9]. The fact that they only observed significant differences between those two groups with regard to the single item “difficulty to smell or taste” but not in the other two above-mentioned items may be due to the fact that no stratification according to gender was performed.

Here, we specifically analysed patients suffering from CRSwNP or AERD in a retrospective evaluation of questionnaires. However, with regard to CRS (comprising both CRSsNP and CRSwNP), Baumann et al. showed in a prospective study that women preoperatively performed worse with regard to QoL score as compared to men [31, 32]. Interestingly, postoperatively no difference between the two gender groups was observed. Similar results were shown by Lal et al. who investigated gender-specific differences in CRS patients electing endoscopic sinus surgery in a retrospective review and reported more problems with postnasal drainage, embarrassment, and facial pain in women preoperatively. In their postoperative analysis, men and women showed similar symptom scores [13]. Since our study was a retrospective evaluation of the questionnaires and independent of the time or number of surgeries, this could be a reason for the non-significant differences in symptoms between the groups. Another limitation of our study is the relatively small sample size of patients with CRSwNP in comparison to other studies. However, it needs to be mentioned that other studies did not split the subgroups of chronic sinusitis with nasal polyps and thus failed to investigate sex-specific differences depending on the presence of polyps and subtypes. By being the first to differentiate the subgroups, this study contributed to understanding differences according to sex in patients suffering from various forms of nasal polyposis.

Future studies should aim at prospectively investigating the differences in sex-specific complaints of patients with CRSwNP and AERD before and after surgical intervention. In light of current developments it would also be interesting to assess patients’ QoL before and after therapy with monoclonal antibodies targeting IgE, interleukins and interleukin receptors, which have shown promising effects in patients suffering from CRSwNP [33].

## Conclusion

This is the first study assessing the differences in QoL between CRSwNP and a large cohort of AERD patients in relation to sex. The aim of this study was to clarify the impact of these diseases on patients’ QoL in men and women to possibly highlight important differences to be taken into account for therapeutic treatment. We did not observe significant differences in TPS or QoL between the study groups.
